# Psychometric evidence of the Acceptance and Action Questionnaire-II (AAQ-II): an item response theory analysis in university students from Chile

**DOI:** 10.1186/s40359-024-01608-w

**Published:** 2024-03-01

**Authors:** Álvaro I. Langer, Fernando P. Ponce, Jorge L. Ordóñez-Carrasco, Reiner Fuentes-Ferrada, Scarlett Mac-Ginty, Jorge Gaete, Daniel Núñez

**Affiliations:** 1 Millennium Nucleus to Improve the Mental Health of Adolescents and Youths, Imhay, Santiago, Chile; 2 Faculty of Psychology and Humanities, Universidad San Sebastián, Valdivia, Chile; 3 Faculty of Psychology, Universidad de Talca, s/n, Talca, Chile; 4 Millennium Nucleus on Intergenerational Mobility: From Modelling to Policy (MOVI), Santiago, Chile; 5 Department of Psychology and Sociology, Universidad de Zaragoza, Zaragoza, Spain; 6 Department of Health Service and Population Research, Institute of Psychiatry, Psychology and Neuroscience, King’s College London, London, UK; 7 Research Center for Students Mental Health (ISME), Faculty of Education, Universidad de los Andes, Santiago, Chile

**Keywords:** Experiential avoidance, Psychometric properties, Item response theory, University students

## Abstract

**Background:**

Experiential avoidance (EA) is a psychological mechanism associated with several mental health disorders and is regarded as a relevant target by third-generation cognitive behavioral therapies. It has been mainly assessed through self-report questionnaires, and the AAQ-II is the most used tool. Its psychometric evidence has been mostly tested through the classical test theory (CTT) and very scarcely assessed through Item Response Theory (IRT).

**Methods:**

We used the Graded Response Model to examine its psychometric properties in Spanish-speaking university students (*n* = 1503; women = 995 (66.2%), mean age = 19.29, SD = 2.45). We tested whether the empirical data fit the model’s predictions and estimated the dispersion of persons and items along the experiential avoidance continuum. Moreover, we examined category probability curves to identify the response probability of each answer. Likewise, an item-person map was made where the measurement of persons and items, both on the same scale and along the experiential avoidance continuum, could be observed jointly. Finally, we tested the gender invariance of the scale.

**Results:**

We found that the values of the individuals and the items were in the established range to be considered an adequate measure of EA. Additionally, we observed high discrimination indices for all items. The current version with seven answer options could not be optimal and should be tested in future studies. Finally, we found evidence of differential functioning by gender in one of the seven items of the instrument.

**Conclusions:**

Our results indicate that the AAQ-II is a suitable tool for measuring EA and accurately classifying and differentiating EA levels in university students.

**Supplementary Information:**

The online version contains supplementary material available at 10.1186/s40359-024-01608-w.

## Introduction

Experiential avoidance (EA), the person’s attempts or desires to suppress unwanted internal experiences, even when this leads to actions that are inconsistent with personal values and goals [1, 2], is regarded as a transdiagnostic process associated with the development and maintenance of a wide range of psychopathological disorders [3]. Recent research with clinical samples has shown associations with depression and anxiety [4], posttraumatic stress symptoms [5], psychotic spectrum disorders [6], substance abuse disorders [7], and suicide experiences and non-suicidal self-injury [8, 9].

EA has also been related to psychopathology in individuals from the non-clinical population [10, 11] like undergraduate students, where mental health difficulties are prevalent [12]. In this population, EA has been linked to different risk factors and mental disorders [13] and could be a potential predictor of depressive and anxiety symptoms [14]. Moreover, EA might moderate the relationship between stigma and help-seeking behaviors for mental health assistance [15] and the relationship between perceived stress and alexithymia symptoms in young students [16].

EA is currently considered a relevant clinical target by contemporary psychotherapeutic approaches such as Acceptance and Commitment Therapy [17]. There is a recognized need to measure this construct accurately [18], which has been mostly addressed through self-report questionnaires [19]. One of the most widely used questionnaires is the Acceptance and Action Questionnaire [AAQ; 2]. The original 10-item version has shown problems concerning its internal consistency and factor structure, which could be associated with unnecessary item complexity [20]. To overcome these issues, a new 7-item version, the AAQ-II, was developed [20]. It has been validated in clinical and general populations from different countries [21–23]. Its psychometric properties have also been explored in university students in countries such as China [24], Turkey [25], the United Kingdom [26], and also from Latin American countries such as Ecuador [27], Perú [28], and Brazil [29]. These studies have reported good psychometric evidence.

Most prior psychometric studies have used the classical test theory, where the test and item statistics (e.g., reliability, discriminative capability) strongly depend on the study samples [30]. This does not occur with the Item Response Theory, which, among other contributions, additionally provides information about the accuracy of a scale to classify the latent trait levels and the capability of every single item to discriminate better the latent trait [IRT; 31]. This could be useful to accurately determine which items discriminate against individuals who could benefit from interventions to reduce EA. Thus, this approach recognizes that a questionnaire’s precision measurement should vary according to the latent attribute values (EA values) instead of assuming a standard estimate for all individuals [32]. The only two previous studies using this approach using the 10-item [33] and the 7-item version [18] suggest that it is a promising method to understand EA better and to increase the knowledge of the performance of the AAQ-II in different populations, which is strongly encouraged nowadays [18]. To our knowledge, no AAQ-II studies from Spanish-speaking Latin-American countries use IRT. Considering this gap, we used these methods to test the performance of the current seven answer options. Through the Graded Response Model [GRM; 34], we examined the item and person measurement using the same measurement scale (i.e., logits). Moreover, we assessed the scale’s reliability and validity evidence (structure and criterion-related). Finally, given the scant evidence on the gender invariance of the AAQ-II and the need to better understand EA in women and men [35, 36] we explored the differential item functioning based on gender.

## Method

The study was approved by the scientific ethical committee of the National Health Service in Valdivia (n° 075) and the Universidad de Talca (03-2021). The study procedures were carried out in accordance with the Helsinki Declaration.

### Transparency and openness

The study protocol was not preregistered. Datasets and scripts generated during and analyzed during the current study are available in the Open Science Framework repository (https://osf.io/43dfq/?view_only=a97186042d6a474aad93880b183935fc).

### Participants

The finite population of interest was composed of all first-grade students from two universities in Chile aged over 18 years (*N* = 5.517). The rate answer was between 29% and 40%. The sample comprised 1,705 university students (66.5% females, M_age_ =19.29, *SD*_*age*_ =2.45) who signed the written and informed consent before completing the survey. This sample size exceeds the recommended size for confirmatory factor and IRT models [37–39]. Missing values on the variables used in this study were minimal (*n* = 13; 0.8%) and not missing completely at random (MCAR), as assessed through Little’s MCAR Test [40; χ^2^ = 81.9; df = 78; *p* = 0.359]. Therefore, due to these results, the large sample size, and the ease of analyses, we employed a pairwise deletion of individuals who did not respond to any item, leaving a sample size of 1,692.

In addition, an inspection of participants’ response patterns using multivariate normality analysis (with the ‘*mvn*’ package; [41]and the detection of potentially careless responses [using the ‘*careless*’ package; 42] showed a small number of participants identified with potentially careless responses and as multivariate outliers (n = 189; 11.1%). These participants tend to introduce error variance that affects the factor structure of the scale, among other consequences [43, 44]. After both examinations, the final sample included 1,503 participants.

The inclusion criteria was being a first-year undergraduate student aged over 18 years. We did not use any exclusion criteria.

### Procedure

All first-year undergraduate students aged over 18 years were invited to participate in the study. Web surveys based on the World Mental Health International College Student Initiative [WMH-ICS; 45] were applied remotely from June to October 2021. The surveys were accessed through Qualtrics software via e-mails and social media links. To access the study, students were required to provide their identification number and surname. Upon access, they encountered a straightforward description of the study’s purpose and provided informed consent by selecting the “yes” option to participate and proceed with the survey voluntarily.

### Measures

#### Acceptance and Action Questionnaire-II [AAQ-II; 20]

The AAQ-II is a 7-item scale designed to assess experiential avoidance (EA). Items were scored on a 7-point Likert-type scale (1 = Never true; 7 = Always true), with higher scores indicating greater levels of EA. We used the Spanish validation of the AAQ-II, as developed [23] and confirmed the comprehensibility of the items in a sample of 20 Chilean university students who did not report comprehension difficulties. In our study, the internal consistency indices (Cronbach’s alpha and the Omega coefficient) suggest excellent reliability (α = 0.94; ω = 0.94).

#### Short Warwick-Edinburgh Mental Well-Being Scale [SWB; 46]

The SWB is a 7-item measure used to assess positive components of mental well-being. Participants were asked to rate items based on their experience over the past two weeks using a 5-point scale (1 = Never; 5 = Always), with a higher score indicating greater mental well-being. We used a short 7-item translated version of the Spanish validation of this scale [47]. This shorter scale showed good internal consistency reliability (α = 0.88; ω = 0.88).

#### Anxious and depressive symptoms

Participants were asked about the frequency of specific thoughts or sensations they had experienced in the past 30 days, using a 5-point scale (1 = None of the time; 5 = All or nearly all of the time). This survey was based on the World Health Organization World Mental Health International College Student initiative [WMH-ICS; 45].

Table 1 shows the descriptive statistics of the variables used in this study, while Supplementary Table 1 shows the frequency statistics for each response category in each AAQ-II item.

**Table 1:** Descriptive statistics for variables used in the study

AAQ-II Items	Descriptives					
	Mean	SD	Min	Max	Skew	Kurt
DEP1. Feel sad or depressed?	2.90	1.04	1.00	5.00	0.14	-0.46
DEP2. Feel discouraged about how things were going in your life?	3.07	1.16	1.00	5.00	-0.04	-0.78
DEP3. Take little or no interest or pleasure in things?	3.00	1.19	1.00	5.00	0.02	-0.84
DEP4. Feel down on yourself, no good, or worthless?	2.98	1.37	1.00	5.00	0.02	-1.20
ANX1. Feel worried or anxious?	3.26	1.08	1.00	5.00	-0.02	-0.70
ANX2. Worry about a number of different things in your life, such as your work, family, health, or finances?	3.28	1.18	1.00	5.00	-0.02	-0.84
ANX3. Feel more worried than other people in your same situation?	2.92	1.23	1.00	5.00	0.10	-0.94
ANX4. Worry excessively or too much?	3.33	1.23	1.00	5.00	-0.25	-0.89
SWB1. I’ve been feeling optimistic about the future.	3.09	1.07	1.00	5.00	0.00	-0.65
SWB2. I’ve been feeling useful.	3.10	1.01	1.00	5.00	0.08	-0.59
SWB3. I’ve been feeling relaxed.	2.55	0.97	1.00	5.00	0.45	-0.20
SWB4. I’ve been dealing with problems well.	3.14	0.98	1.00	5.00	-0.08	-0.45
SWB5. I’ve been thinking clearly.	3.15	0.98	1.00	5.00	-0.04	-0.46
SWB6. I’ve been feeling close to other people.	3.35	1.09	1.00	5.00	-0.19	-0.76
SWB7. I’ve been able to make up my own mind about things	3.91	0.97	1.00	5.00	-0.64	-0.24
AAQ1. My painful experiences and memories make it difficult for me to live a life that I would value.	3.04	1.75	1.00	7.00	0.55	-0.70
AAQ2. I’m afraid of my feelings.	3.39	1.79	1.00	7.00	0.24	-0.99
AAQ3. I worry about not being able to control my worries and feelings.	3.91	1.89	1.00	7.00	-0.01	-1.12
AAQ4. My painful memories prevent me from having a fulfilling life.	2.75	1.82	1.00	7.00	0.85	-0.36
AAQ5. Emotions cause problems in my life.	3.37	1.83	1.00	7.00	0.35	-0.90
AAQ6. It seems like most people are handling their lives better than I am.	3.67	2.00	1.00	7.00	0.16	-1.21
AAQ7. Worries get in the way of my success.	3.53	1.85	1.00	7.00	0.25	-1.200

**Note. Items**: DEP = Depression symptoms; ANX = Anxiety symptoms; SWB = Subjective well-being; AAQ = Experiential avoidance. **Descriptives**: SD = Standard deviation; Min = Minimum; Max = Maximum; Skew = Skewness; Kurt = Kurtosis.

### Data analysis

The data were analyzed using the Graded Response Model [GRM, 34, 48, 49] with the package ‘*mirt*’ [50]. Beyond the standard assumptions of IRT, the GRM assumes that the response categories to which individuals respond or qualify can be ordered on a hierarchy, often represented using probabilistic scales for summation estimates. This model is essentially an extension of the two-parameter logistic model (2-PLM) designed to accommodate multiple ordered categories and is categorized among ‘difference models’ [51]. These models are typically considered as indirect IRT models due to the two-step process required for calculating conditional probabilities for specific response categories.

The GRM model defines the likelihood of a responder meeting the criteria for an item *i* with a category response option equal to or higher than *k* rather than being included in a lower category *k– 1*, assuming a rating system comprised of at least three categories. This model can be expressed as follows:

1 $$ {P}_{ik}^{*}\left({\theta }_{j}\right)=\frac{{e}^{{D}_{{\alpha }_{i}}({\theta }_{j}-{\beta }_{ik})}}{1+{e}^{{D}_{{\alpha }_{i}}({\theta }_{j}-{\beta }_{ik})}}$$

Where P^*^_ik_(θ_j_) denotes the probability that a responder with a definite latent trait or ability level *θ*_*j*_ endorses a response category *k* or above in a given item *i*, α_i_ is the discrimination parameter of item *i*, and *β*_*ik*_ is the item localization (threshold) of item *i* on response category *k*, and D is the constant scale factor for the logistic function (D = 1.702).

Then, by subtracting the cumulative probability to the right of a given category and that of the next one, it is possible to obtain P_ik_(θ_j_), which denotes the probability of endorsing a specific response category *k* of item *i* given a latent trait level *θ*_*j*_.

2 $$ {P}_{ik}\left({\theta }_{j}\right)={P}_{ik}^{*}\left({\theta }_{j}\right)-{P}_{ik+1}^{*}\left({\theta }_{j}\right)$$

We selected the Graded Response Model over alternative models such as the Partial Credit Model [52, 53] or the successive intervals model [54] because it was designed to analyze polytomous ordinal items with varying discrimination parameters. This suitability extends to estimating cumulative sum scales, similar to those commonly found in Likert-type scales.

To ensure that the calibration is appropriate, the items must meet the assumptions of an IRT model (unidimensionality, local independence, and monotonicity), and, secondly, the theoretical model must fit the empirical data.

Unidimensionality was evaluated using Confirmatory Factor Analyses (CFA) using maximum likelihood with robust standard errors (MLR) as an estimation method. The goodness of fit of the estimated models was evaluated using the comparative fit index (CFI), the Tucker–Lewis fit index (TLI), the root mean square error of approximation (RMSEA), and the standardized root mean square residual (SRMR). For the CFI and TLI indices, estimated values above 0.90 and 0.95 indicate an acceptable and good fit level, respectively [55, 56]. For the RMSEA and SRMR indices, values equal to or less than 0.05 and 0.08 are considered good and acceptable, respectively [55, 57]. The analyses were conducted using the package ‘*lavaan*’ [58].

Local independence refers to whether the items are exclusively linked to the measured primary construct and remain unaffected by other factors. This implies that, once the influence of the primary factor is accounted for, there should be no noteworthy covariation among item responses. To assess local independence, we used the function ‘*localdep*’ [59] and then examined the residual correlation matrix derived from a single-factor CFA model. In line with prior research [60], a critical threshold for local dependence was set at 0.20 above the average residual correlation.

Monotonicity examines whether the probability of an affirmative response to the items increases with increasing levels of the underlying construct. It was evaluated by fitting a non-parametric IRT model through Mokken scaling using the package *‘mokken’* [61, 62] and calculating the scalability coefficient H per item and for the total scale. H values equal to or less than 0.30 and 0.50 are considered acceptable for items and the whole scale, respectively [61].

Model fitting was judged by examining the ease of convergence (number of iterations) for estimating the model parameters, reasonable parameter estimations, and standard error of parameters. In addition, it was examined using the limited-information goodness of fit test statistic C2 for ordinal IRT models [63]. Unlike the M2 [64] and M2* [65] statistics, this statistic can be computed even in scenarios when the number of items is small, and the number of categories is large, as in the situation here. In addition, M2-based root mean square error of approximation (RMSEA), standardized root mean square residual (SRMR), and comparative fit index (CFI) were used to assess the adequacy of model fit.

In order to assess how well each item fits the model, we used the index S-X^2^ [66, 67], computed from the RMSEA value that serves as a metric for gauging the extent of item fit. Values less than 0.06 indicate a satisfactory item fit. Additionally, we analyzed the infit (inlier-pattern-sensitive fit) and outfit (outlier-sensitive fit) statistics by estimating mean square fit statistics (MNSQ). In both cases, MNSQ values between 0.5 and 1.5 can be considered indicative of an adequate fit [68].

Once we established the adequacy of the model and item fits, we computed item parameters to assess item-latent trait association, and category probability curves were examined [69]. Specifically, these curves allow identifying the response probability for each category (in this case, Likert-type response options of AAQ-II items) as a function of responders’ trait level. The GRM model yields two item types of parameter estimates: the item slope and item thresholds [70]. The item slope parameter (α_i_) refers to the discriminative ability of the items, with higher slope values indicating a stronger association with the trait level. Item threshold (or localization) parameters (β_ik_) locate item response categories along the trait level. For items with seven response categories, six thresholds were estimated.

In the context of IRT, measurement precision is conceptualized as the “information” (I) that the test and items provide, which can vary (is a *conditional* index) across the range of the measured trait or ability (I|θ_j_). Each estimated *I* value has an associated standard error (SE) that also varies at each point of theta (SE|θ_j_). Conditional reliability coefficients can be derived from information values from simple transformations [71].

For instance:

3 $$ \rho (X,{X}^{{\prime }}|\theta )=\frac{I\left(X\right|\theta )}{\left[I\left(X\right|\theta )+1\right]}$$

where ρ(X,X’|θ) is the conditional reliability of X at a fixed value of θ, and I(X|θ) is the score information function. This equation reflects the simple relationship between these primary indices of measurement precision in IRT. Conditional reliability allows for identifying the specific subgroups within the trait range for which a test precisely measures and, conversely, identifying those subgroups where the test produces unreliable scores. Another application of conditional reliability involves estimating a test or item score’s overall (or marginal) reliability. This can be done by integrating the conditional reliabilities to obtain a marginal score estimate [72].

Subsequently, we used the model parameters to compute participants’ theta scores (person parameters) using the expected a posteriori [EAP; 73]procedure. The EAP method was utilized to generate the scores, transform those estimates into the original scale metric, and depict them in a scale characteristic function curve. This function provides a more familiar reference for interpreting scores. In our case, expected true scores refer to scores on the AAQ-II scale metric (7 to 49) that are expected as a function of estimated participant theta scores.

Differential item functioning (DIF) was examined using the log-likelihood ratio test method [LRT; 74]with the function ‘multipleGroup’ [50] to handle group differences in trait distributions with gender as the grouping variable [75]. Our analytical approach followed a sequential specification of analyses that incorporated more restrictive models by imposing parameter constraints on the model structure, discrimination, difficulties, and means parameters of both groups. Differential item functioning was established once a significant detriment was identified with the chi-square difference test.

Finally, we used the EAP-generated scores to analyze the distribution of persons’ scores throughout the theta range, comparing them with the estimated parameters for each AAQ-II item through the Wright map.

## Results

### Unidimensionality, local independence, and monotonicity

For the single-factor model, Confirmatory Factor Analysis yielded unsatisfactory fit indices (χ^2^ = 869.991, df = 14, *p* < 0.001, CFI = 0.894, TLI = 0.842, RMSEA = 0.202, 95% CI [0.191, 0.213], SRMR = 0.041). Previous research has suggested the necessity of correlating the uniquenesses of items 1 and 4, as well as items 2 and 3, due to the content overlap between those items [20, 21, 76]. All the fit indices improved, suggesting that the modified model fit the data better (χ^2^ = 146.506, df = 12, *p* < 0.001, CFI = 0.983, TLI = 0.971, RMSEA = 0.086, 95% CI [0.075, 0.099], SRMR = 0.017).

In terms of local dependence, four (19%) of the residual correlations were positive. The average residual correlation was − 0.079, so the critical value 0.20 above the mean would be 0.12 [60]. Three residual correlations (out of 21, 0.14%) were larger than 0.12, suggesting certain local dependence between pairs of items. This apparent lack of local dependence is consistent with the pair of correlated uniqueness reported due to overlapping content between pairs of items. Therefore, both results are consistent in evidencing the existence of a clearly dominant factor but not “pure” unidimensionality.

Finally, regarding the notion of monotonicity, the scalability coefficients (Hi) for the individual items spanned from 0.689 (AAQ6 ‘It seems like most people are handling their lives better than I am’) to 0.766 (AAQ5 ‘Emotions cause problems in my life’), as detailed in Table 2. The overall Mokken scalability coefficient (H) for the entire item bank was calculated at 0.731 (SD = 0.009). Hence, it can be concluded that the AAQ-II items satisfactorily met the monotonicity assumption.

**Table 2:** Fit indices, discrimination (α_i_), and localization (β_ik_) parameters for AAQ-II items

					Items			
		AAQ1	AAQ2	AAQ3	AAQ4	AAQ5	AAQ6	AAQ7
Monotonicity	Scalability coefficient H_i_	0.729 (0.011)	0.725 (0.010)	0.742 (0.009)	0.727 (0.011)	0.766 (0.008)	0.689 (0.012)	0.743 (0.009)
Fit Indices	S-Χ^2^	126.592	107.409	88.265	119.623	84.103	154.272	112.271
	df	101	100	97	113	86	120	99
	*p*-value	0.043	0.288	0.725	0.317	0.538	0.019	0.171
	RMSEA S-X^2^	0.013	0.007	<0.001	0.006	<0.001	0.014	0.009
Parameters	α_i_	2.845 (0.128)	2.957 (0.125)	3.206 (0.130)	2.878 (0.133)	4.096 (0.177)	2.516 (0.106)	3.357 (0.138)
	β_i1_	-0.851 (0.043)	-0.978 (0.045)	-1.207 (0.049)	-0.464 (0.039)	-0.886 (0.040)	-1.049 (0.049)	-1.024 (0.044)
	β_i2_	-0.096 (0.036)	-0.353 (0.037)	-0.585 (0.038)	0.173 (0.037)	-0.292 (0.034)	-0.426 (0.040)	-0.357 (0.036)
	β_i3_	0.258 (0.038)	0.024 (0.036)	-0.215 (0.035)	0.534 (0.040)	0.092 (0.034)	-0.055 (0.038)	0.006 (0.035)
	β_i4_	0.944 (0.047)	0.707 (0.041)	0.357 (0.037)	1.055 (0.050)	0.693 (0.039)	0.483 (0.041)	0.637 (0.039)
	β_i5_	1.522 (0.062)	1.289 (0.054)	0.913 (0.044)	1.487 (0.061)	1.216 (0.047)	0.993 (0.049)	1.186 (0.049)
	β_i6_	2.157 (0.086)	2.027 (0.078)	1.572 (0.060)	1.963 (0.077)	1.730 (0.062)	1.603 (0.066)	1.751 (0.065)

Note. AAQi = Acceptance and Action Questionnaire item. **Fit indices**: S-X^2^ = Orlando and Thissen’s Pearson S-X^2^ statistic; df = degrees of freedom; RMSEA = root mean square error of approximation. Numbers in parentheses are standard error for estimates

### IRT Model and items fit

The calibration was conducted using a discrimination parameter (α_i_) and six (*k*-1) localization parameters (β_ik_) for each of the seven items (Table 2). Parameterization was reached through 112 iterations, resulting in reasonable estimations and good standard error values. For discrimination parameters, the standard error of the measurement (SE) ranged from as low as 0.106 for item AAQ6 (α_i=6_) to 0.177 for item AAQ5 (α_i=5_). As for item difficulties, the SE ranged from 0.034 for item AAQ5 (β_i=5,k=2_) to 0.086 for item AAQ1 (β_i=1,k=6_). Therefore, we conclude that the SE values were sufficiently reduced to suggest a good data fit.

Conversely, the C2 statistics obtained for the scale (C2 = 728.829; df = 14; *p* < 0.001; RMSEA = 0.181) indicate a lack of fit. However, the associated RMSEA values might be because of the limited amount of “modeled error” and the high number of response categories used here [63]. This relationship creates challenges for interpreting RMSEA. Indeed, CFI and TLI values were around the recommended 0.95 threshold (CFI = 0.955; TLI = 0.932), and the SRMR value was equal to 0.052, leading us to conclude that the data fit might be sufficient if one considers the rest of the indices obtained. These results support the appropriateness of GRM to the data.

**Fig. 1:**
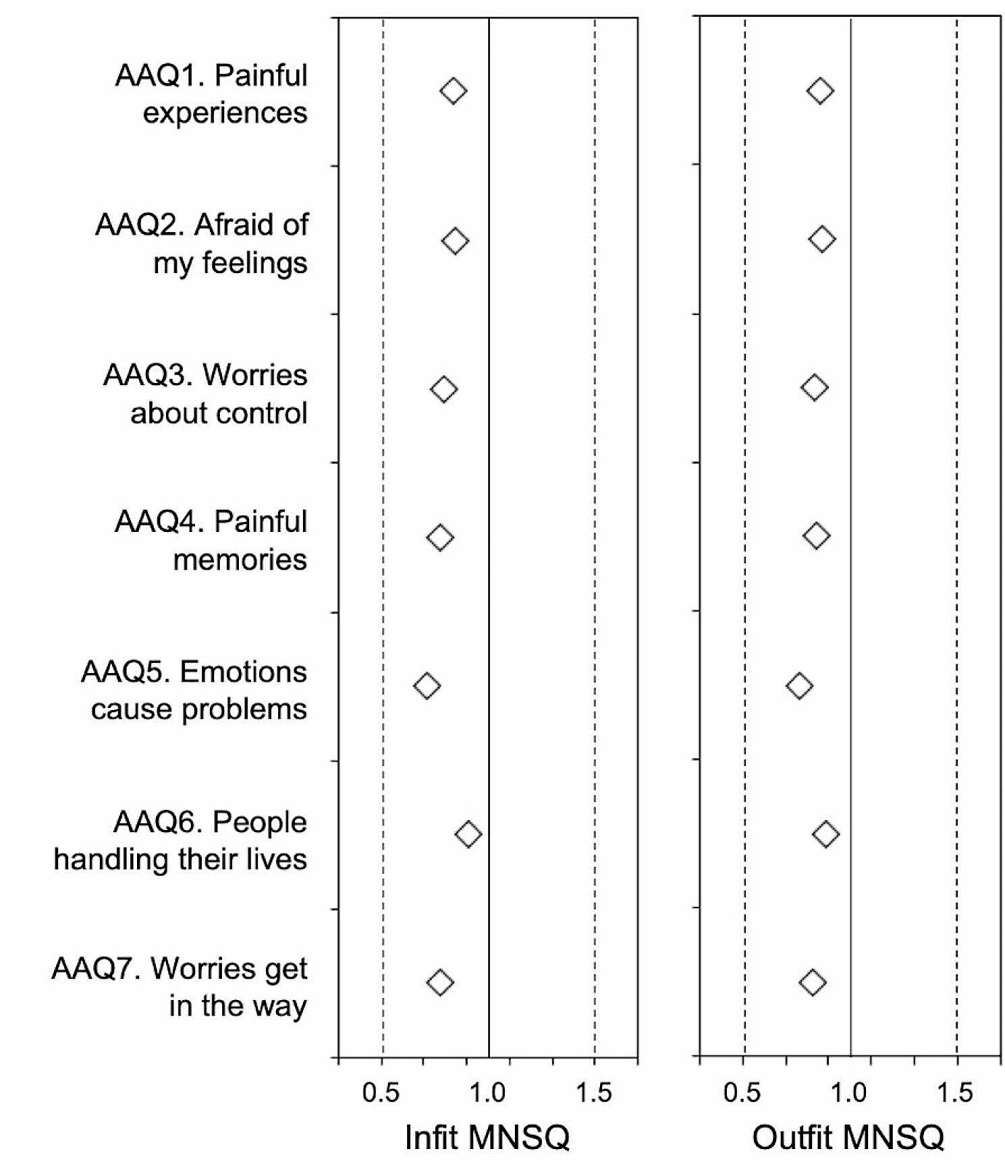
AAQ-II items infit mean square (left) and outfit mean square (right) values. **Note.** Items with values within 0.5 and 1.5 are considered to be productive for measurement

In terms of item fit, a graphical representation of the item fit by infit and outfit MNSQ is provided in Fig. 1. All items were located in the area of 0.5 to 1.5 (areas of acceptable fit), showing their usefulness for measurement [77, 78].

### Parameter estimation

**Fig. 2:**
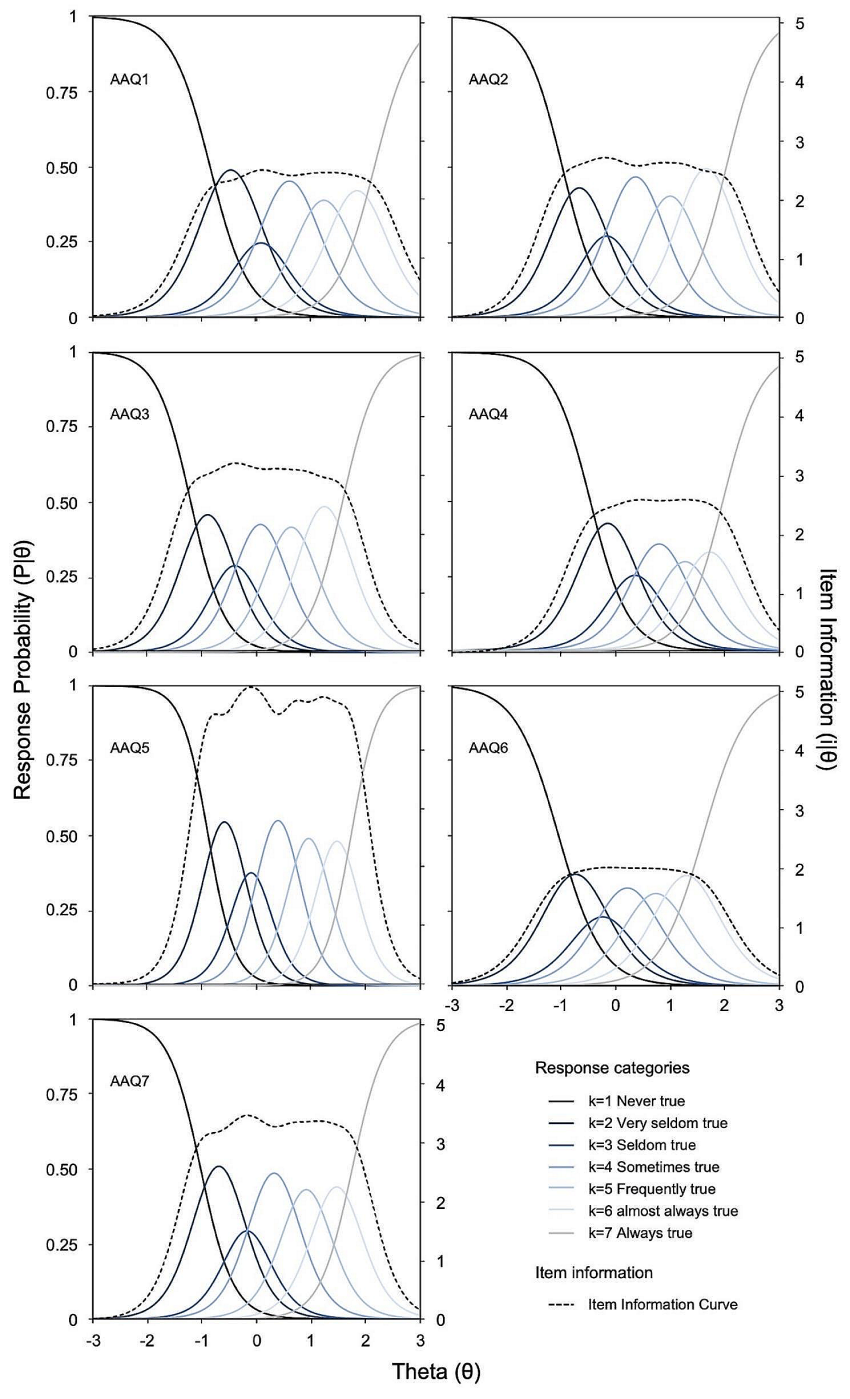
Item characteristic response and information curves for AAQ-II items

To estimate the α_i_ and β_ik_ parameters for the items, the marginal maximum likelihood method was used. Figure 2 depicts the characteristic response curves for AAQ-II items. The horizontal axis represents the latent variable θ (Mean = 0; SD = 1). Seven curves were drawn for each item, each representing the relationship between the attribute level and the probability (defined on the vertical axis) of endorsing one of the seven response categories used.

Table 2 also displays the AAQ-II items parameterization according to the GRM. The item discrimination parameters ranged from α_i=6_ = 2.516 to α_i=5_ = 4.096. The item with the lowest discriminative ability was AAQ6 (‘It seems like most people are handling their lives better than I am’), while AAQ5 (‘Emotions cause problems in my life’) was the item with the highest discriminative ability. In accordance with Baker’s categorization [79], discrimination patterns were very high for all items, indicating steeper slopes and better at differentiating theta. This result was confirmed by the high correlation between parameters α_i_ and the corrected item-total correlations (*r* = 0.917).

In terms of β_ik_ parameters, the values spanned from β_ik=1_ =-1.207 to β_ik=6_ = 2.157 SDs, indicating that responses covered a wide range of the latent trait. The most significant increase was observed between β_ik=1_ and β_ik=2_, ranging between 0.594 and 0.755. Conversely, the interval between β_ik=2_ and β_ik=3_ was narrower, ranging between 0.354 and 0.384. Taken together, β_ik=1_ values are situated approximately at 1SD below the mean (Mean βs =-0.923), while β_ik=2_ and β_ik=3_ values are roughly located at the mean of the latent variable (Mean βs =-0.277 and 0.092, respectively). As for β_ik=4_ and β_ik=5_, they were situated about 1SD above the mean (Mean βs = 0.697 and 1.229, respectively), and β_ik=6_ values are located at 2SDs (Mean βs = 1.829) above the mean.

For a more visual understanding, Fig. 2 provides a graphical depiction of the association between an individual’s ‘ability’ or ‘trait’ and how it influences their responses on the AAQ-II scale. In line with the model’s expectations, each of the categories was the most probable at some point along the latent variable, except for the k = 3 response category, “Seldom true,” which exhibited lower selection probabilities than adjacent response categories on four items (AAQ1, AAQ2, AAQ4, and AAQ6, as depicted in Fig. 2). This result is consistent with the mentioned slightest increment between parameters β_ik=2_ and β_ik=3_. From this perspective, item AAQ5 (“Emotions cause problems in my life”) showed better functioning with ordered and adequately separated response probabilities, including leptokurtic distributions for each response category. This pattern observed in item AAQ5 explains the fact that it was the item that yielded an item information curve with higher values of the items that comprise the scale.

In this respect, the dotted line in Fig. 2 depicts the item information curve, which shows the range of ability levels above θ where the item better distinguishes among individuals being assessed. In other words, it characterizes the precision of measuring individuals at different levels of the latent construct, with higher values indicating increased accuracy. For instance, consider item AAQ6 (“It seems like most people are handling their lives better than I am”), which begins to offer the maximum information for individuals at θ = 1SD and then sharply decreases beyond θ = 1.8SDs. Within this latent variable range, this item becomes most useful and similar to that covered by item AAQ5, but whose magnitude of information and capacity to distinguish is notably lower than the latter. Both items enable us to illustrate how the magnitude and distribution of response category probabilities impact the amount of information the item provides to assess the trait.

### Measurement precision

**Fig. 3:**
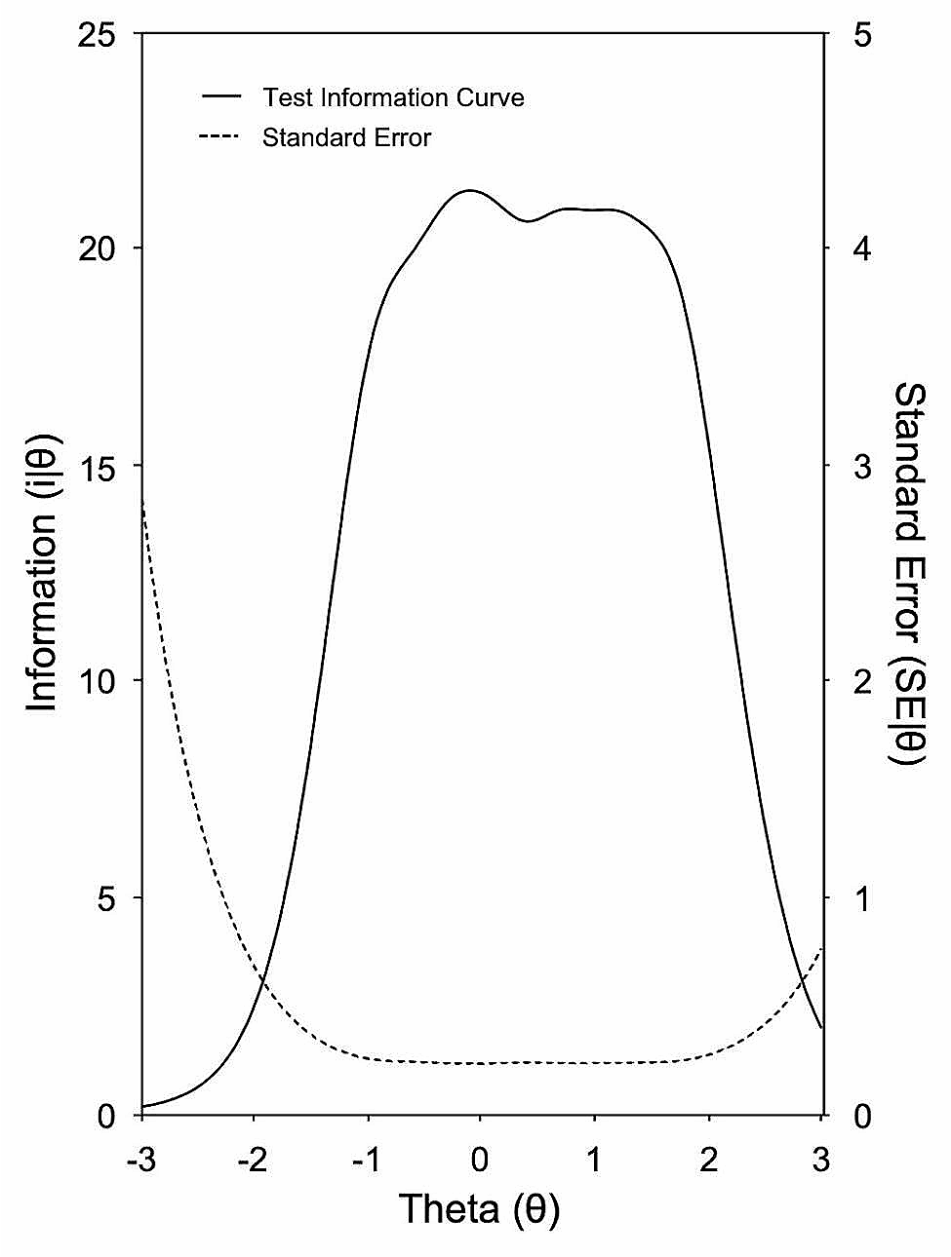
Test information function and standard error of measurement of the AAQ-II scale

The solid line in Fig. 3 depicts the graphical representation of the test information function (TIF), equivalent to the combined value of the information functions for the seven items). The test information values are greater for θ values between − 0.5 and + 1.5 SDs (i.e., 20.33 and 20.38). The lowest standard error values (dotted curve in Fig. 3) correspond to those with the greatest test information. As expected, the smaller SE values denote more information or precision in the scale with regard to latent θ.

**Fig. 4:**
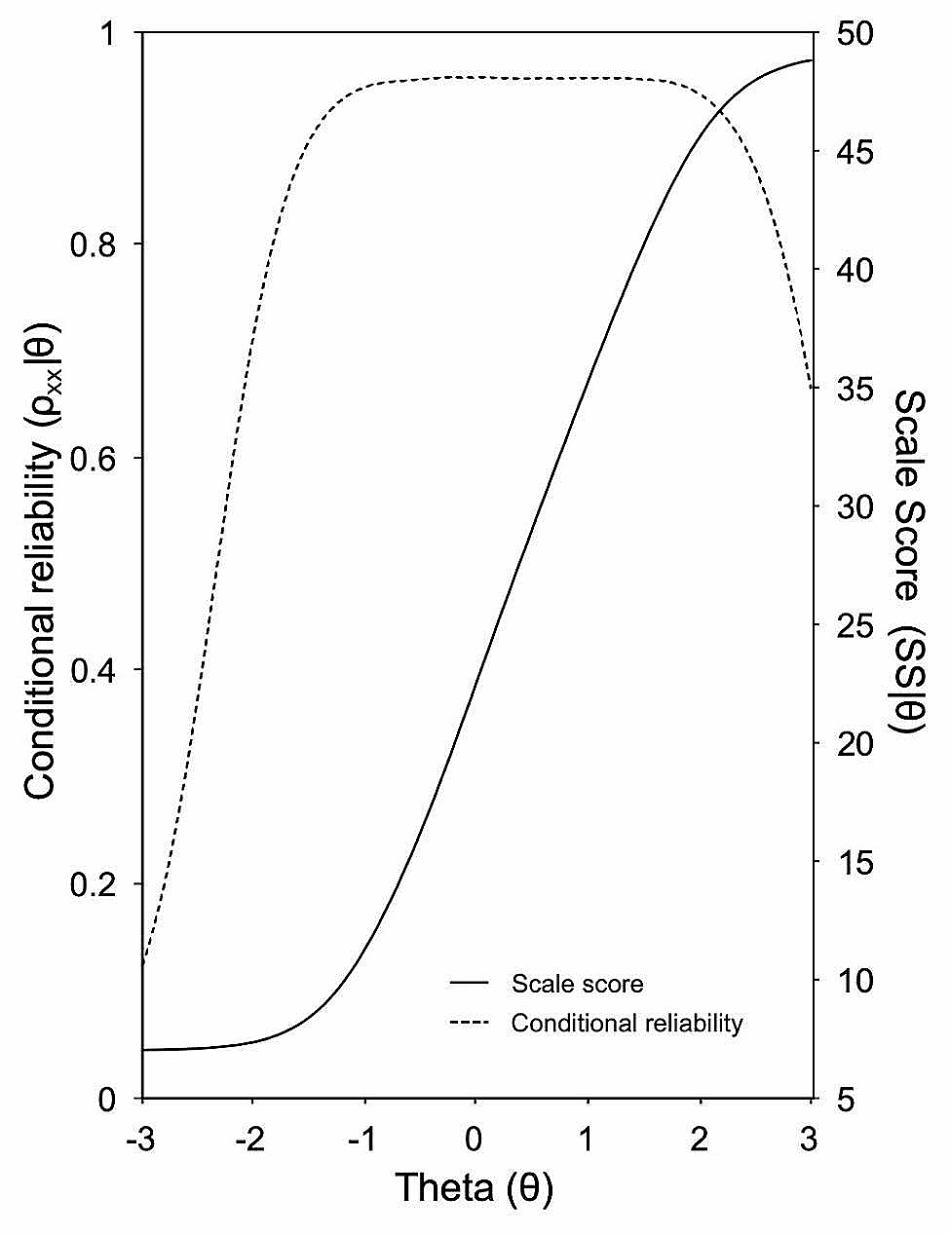
Characteristic test curve and conditional reliability estimates of the AAQ-II scale

The AAQ-II scale reaches its maximal accuracy between about the mean and a relatively high region of the latent variable. The discriminative capability of scores decreases very fast in the higher areas of the trait. This greater degree of precision of the scale measuring the attribute in high attribute regions was also found when examining the characteristic test curve (CTC) and conditional reliability (both depicted in Fig. 4). For the scale score values, the CTC function predicted a noticeable quantity of latent trait above the mean (θ = 0; x = 22.5), suggesting that the most significant variability and their maximal accuracy are obtained at attribute levels above the average. These results were also observed in terms of conditional reliability.

**Fig. 5:**
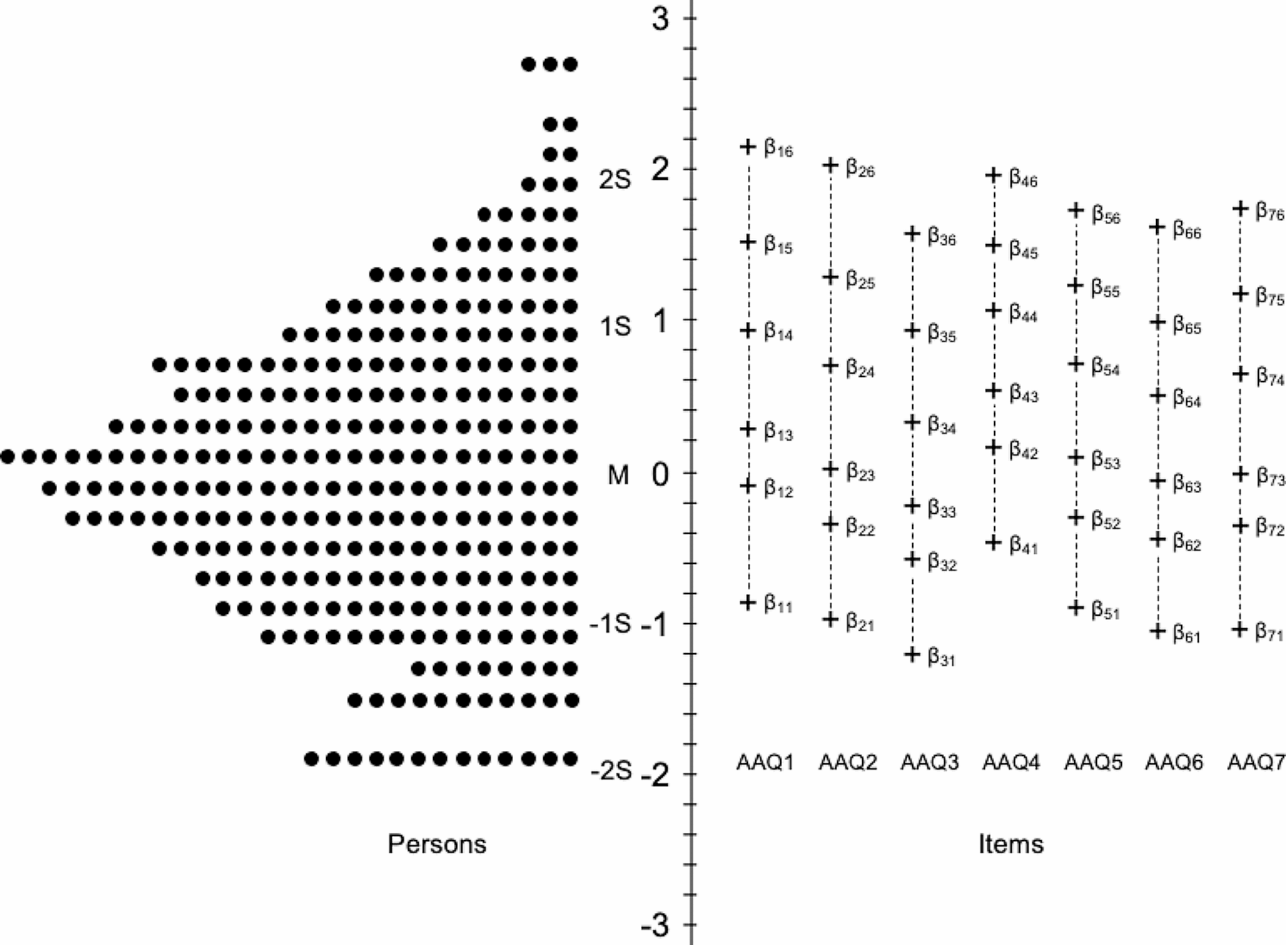
Wright Map (persons and items distributions). **Note.** M = Mean; S = Standard deviation

The distribution of individuals’ scores on the AAQ-II (Me = 0.001; SE = 0.967) is shown on the left side of the Wright map depicted in Fig. 5. This visual representation provides an overview of scores’ variability observed among participants along the theoretical continuum. Additionally, the right side of Fig. 5 illustrates the AAQ-II items according to their degree of severity (Me = 0.441, SE = 0.960).

Collectively, the items cover a wide range along the theoretical continuum, exhibiting a propensity towards higher trait levels and spanning approximately from − 1.2 (AAQ3) to 2.2 (AAQ1). This aligns with the orderly distribution of participants’ scores noted above theta = 0, as opposed to the pattern identified among respondents exhibiting lower levels of Experiential Avoidance (see left side of Fig. 5). Regarding individual items, AAQ3 (“I worry about not being able to control my worries and feelings”) was situated at the lowest point of the experiential avoidance continuum, ranging from − 1.207 to 1.572. Consequently, this item exhibited the lowest range of difficulties in covering response categories. Conversely, AAQ1 (“My painful experiences and memories make it difficult for me to live a life that I would value”) and AAQ4 (“My painful memories prevent me from having a fulfilling life”) tended to discriminate individuals with higher levels of Experiential Avoidance. Specifically, AAQ1 yielded the highest observed threshold (at 2.157), while AAQ4 collectively exhibited higher points than the remaining items. This pattern suggests that these items are particularly effective at discerning individuals with heightened tendencies toward experiential avoidance.

### Differential item functioning

The configural model showed a favorable statistical fit for both groups (AIC = 30,425.170; BIC = 30,946.070; adjBIC = 30,634.750; see Table 3), confirming the findings previously obtained from the entire sample. Likewise, the model, with restricted slopes in its specification, showed a comparable fit to the structural solution (AIC = 30,422.760; BIC = 30,905.930; adjBIC = 30,616.850), suggesting the absence of a statistically significant detriment. The chi-square test confirmed this lack of detriment (X^2^ = 11.073; df = 7; *p* = 0.135).

**Table 3:** Differential item functioning statistics

	Fit indices						Log-likelihood ratio test			
Model	C2	df	AIC	BIC	adjBIC		LogL	X^2^	df	*p*-value
Configural	748.580^***^	28	30,425.170	30,946.070	30,634.750		-15,114.590			
Slopes	754.143^***^	35	30,422.250	30,905.930	30,616.850		-15,120.123	11.073	7	0.135
Intercepts	808.608^***^	75	30,395.820	30,666.900	30,504.890		-15,146.910	53.576	40	0.074
Intercepts and Means	856.858^***^	77	30,444.760	30,705.210	30,549.550		-15,173.380	52.938	2	< 0.001

**Note. Fit indices**: C2 = Limited-information goodness of fit test statistic; df = degrees of freedom; AIC = Akaike Akaike Information Criterion; BIC = Bayesian Information Criterion; adjBIC = Sample size adjusted BIC. **Log-likelihood test**: LogL = Log-likelihood; X^2^ = Chi-square.

On the other hand, a notable detriment was noted in the fit under the solution that sets the difficulty parameters equal between both groups (AIC = 30,395.820; BIC = 30,666.900; adjBIC = 30,504.890). Nevertheless, this difference was only marginally significant upon scrutiny with the chi-square test (X^2^ = 53.576; df = 40; p = 0.076), implying a potential existence of differential item functioning at the difficulty parameter level for men and women. This possibility was explored by examining the presence of uniform DIF in each item separately using the ‘*DIF’* function with the package ‘*mirt*’ [50] by dropping constraints across groups. This analysis revealed a statistically significant misfit (X^2^ = 24.817; df = 6; *p* < 0.001) in the estimated parameters in AAQ3 (“I worry about not being able to control my worries and feelings”). An inspection of parameter values for both groups (see Supplemental Table 2) revealed that women consistently exhibited lower difficulty values. In our sample, after imposing equivalence in the means of both groups, the model’s detriment was statistically significant (X^2^ = 52.938; df = 2; *p* < 0.001), indicating differences between men and women’s means.

### Criterion-related validity

To examine the criterion validity of the AAQ-II scale, correlation with other relevant measures was computed using the individual estimated trait scores derived from the GRM model. The results indicated a strong criterion validity for the AAQ-II, as correlations with all measures were substantial (|rs| >= 0.50; see Table 4). First, a negative correlation (*r* = − 0.642; *p* < 0.001) with mental well-being. Conversely, the AAQ-II exhibited a strong positive association with both anxiety (*r* = 0.604; *p* < 0.001) and depression symptomatology during the last month (*r* = 0.655; *p* < 0.001).

**Table 4:** Correlations of the AAQ-II with criterion-related variables

	AAQ-II	SWB	ANX	DEP
AAQ-II	-			
SWB	-0.642^***^	-		
ANX	0.604^***^	-0.523^***^	-	
DEP	0.655^***^	-0.685^***^	0.710^***^	-

Note. AAQ-II = Acceptance and Action Questionnaire; SWB = Short Warwick-Edinburgh Mental Well-Being Scale; DEP = Depression symptoms; ANX = Anxiety symptoms;

## Discussion

This is the first study examining the psychometric properties of the Spanish version of the AAQ-II using a Graded Response Model [34] in a large sample of first-year university students. Our results indicate that the AAQ-II is sufficiently sensitive to identify different levels of EA. Moreover, we found that the discriminative analysis of each item is adequate. These results fit with prior evidence showing that the scale is a reliable tool to assess the latent trait of EA [36]. We additionally assessed the differential item functioning and we found that there were not significant differences, except for the item AAQ3. Finally, the results about the construct-related validity support prior findings revealing positive associations with psychiatric symptoms [5]. ) and negative relationships with mental wellbeing [80].

The test information function showed that the scale accurately detected the latent trait at each continuum level, particularly within intermediate to high values along the latent trait. This is consistent with the IRT approach, where the precision measurement of a questionnaire should vary according to the latent attribute values (EA values) instead of assuming a common estimate for all individuals [32]. It seems that this approach it is sensitive to capture the nature of the EA construct. In this regard, to avoid inner events classified by each person as stressful or negative could be considered a common and expected psychological reaction [1]. However, when the avoidance becomes a rigid response pattern that goes against what is valuable to a person in life it should be considered psychopathological relevant [81, 82]. Although, more research is needed, this result supports that this scale is suitable to discriminate different levels of EA in university students.

The overall discriminative ability was good for all items. However, some differences were observed. For instance, the item AAQ6 (‘It seems like most people are handling their lives better than I am’) showed the lowest discriminative ability, which was also observed by [18]. By contrast, it was highest for the item AAQ5 (‘Emotions cause problems in my life’). Similarly, Menéndez-Aller et al., [36] using a CTT approach, found that the item 6 presented the lowest discrimination indices in comparison with item 5 which present the highest discrimination values in a Spanish general sample. This item’s differential ability could be potentially explained considering their contents. While the item AAQ6 represents a general evaluation of a life situation, the item AAQ5 is directly associated with stressful inner experiences, which fits better with the AE construct.

Regarding the study of response categories, the curves showed that all response options had a greater probability of being selected at some point of the continuum of EA, except for response option 3 (“seldom true”). This suggests an inconsistent response pattern in that response category. Future studies should examine versions with fewer response options.

The distribution of persons in the Wright item-person map, showed that persons were primarily located in the intermediate range of the continuum of EA. This is consistent with prior research showing normal distribution of EA in the general population [83]. It was also possible to observe a group of individuals that are not adequately characterized because their response options do not cover the lower latent trait levels where those individuals were located within the EA continuum. This is consistent with the finding about the higher precision of the AAQ-II to detect EA at a higher level of the latent trait. Concerning the item distribution, the item requiring the lower severity for being endorsed was the AAQ3 (“I worry about not being able to control my worries and feelings”) and the items requiring higher severity for being endorsed correspond to those inquiring about painful experiences and memories (AAQ1 & AAQ4). When comparing the content of these items, it is possible to argue that these two later items represent a higher psychopathological meaning being a potential source of higher distress [84].

The global examination of the differential item functioning yielded no significant differences in the estimated parameters for women and men. This fits with studies showing gender invariance in university students [27, 85–87]. However, a further inspection showed differences in the AAQ3 (“I worry about not being able to control my worries and feelings”) revealing that women consistently exhibited lower difficulty values. Therefore, at the same level of EA, a woman tends to mark a response option associated with a higher score than a man in this item. This does not fully support previous IRT studies revealing that none of the items showed differential item functioning in adults from the general population [33, 36]. On the other hand, our result showing differences between men and women’s means at the latent trait level mirror the evidence showing higher EA levels in women [88], but contrasts the studies showing no significant differences [33, 89]. Given the different age-ranges and questionnaires; 10-version items in the study by Fledderus et al. [33], direct comparisons with our study must be interpreted with caution. Because of the inconclusive evidence about the gender differences in EA, further research is needed to examine the differential item functioning and gender differences in other populations.

The analysis of the fit of the data to the model showed that the values of the items are in the established range to be considered adequate to the requirements of the model [77, 78] as well as evidence of the unidimensionality of the measure [90]. Both results are consistent with evidence showing the existence of a clearly dominant factor but not “pure” unidimensionality. These findings support recent research revealing a three-dimensional structure [91] which requires further research.

### Implications

A previously reported, we found positive associations between EA and anxiety and depressive symptoms [4, 11, 26, 33] and negative correlations with mental well-being [92]. We additionally observed that the maximal accuracy of the scale is obtained above the average of the latent trait, which suggests that, at least in this kind of sample, the scale is adequate to identify individuals using EA as psychological mechanism to cope with inner events. Additionally, the finding that the item AAQ4 “my painful memories prevent me from leading a full life” was the item located highest on the EA continuum, fits with prior research showing associations between EA and traumatic-related experiences [93]. This item may be highly informative about this association orienting the clinical assessment process. Overall, these findings support the potential usefulness of the scale to assess psychotherapeutic and clinical outcomes in university students, particularly those being treated by third generation cognitive-behavioral therapies [94].

This study has some limitations. First, we used a convenience sampling method in first-year university students; therefore, the representativeness is not guaranteed. Considering that different results could be obtained in different samples [18], future studies assessing the differential functioning of the items in other populations (i.e., clinical samples, adolescents, and adult samples) are needed. Second, the focus was the assessment of EA. Therefore, specific theoretical aspects of the construct were beyond the scope of the study, which should be addressed in future research. For instance, exploring the cognitive and or affective nature of the construct, and its adaptive or maladaptive role, and the specific associations with different mental disorders are relevant questions needing further research more in line with a contextual-functional perspective of the construct. This could be achieved using contemporary and complementary methods such as ecological momentary assessment which provides information about fluctuations of psychological and emotional processes in short periods of time [81, 95]. Considering that the AAQ-II is a brief questionnaire, it could be easily combined with other variables using this approach.

In summary, our study supports an adequate psychometric functioning of the Spanish version of the AAQ-II to measure experiential avoidance and to classify and differentiate EA levels in university students. This suggests that in this sample the scale is adequate for screening purposes. Potential improvements for the scale could be obtained by testing psychometric properties using fewer response options.

### Electronic supplementary material

Below is the link to the electronic supplementary material.

Supplementary Material 1

## Data Availability

Datasets and scripts generated during and/or analyzed during the current study are available in the Open Science Framework repository (https://osf.io/43dfq/?view_only=a97186042d6a474aad93880b183935fc).
