# Supplementary material for: Psychometric evidence of the Acceptance and Action Questionnaire-II (AAQ-II): an item response theory analysis in university students from Chile
Source: BMC Psychol. 2024 Mar 1;12:111. doi: 10.1186/s40359-024-01608-w (PMC10908082; doi:10.1186/s40359-024-01608-w)
Supplement: Supplementary file 1 — Supplementary Material 1 [file 40359_2024_1608_MOESM1_ESM.docx]

**Supplemental Material**

Psychometric evidence of the Acceptance and Action Questionnaire-II (AAQ-II):

An Item Response Theory analysis in university students from Chile.

Álvaro I. Langer, Fernando P. Ponce, Jorge L. Ordóñez-Carrasco, Reiner Fuentes-Ferrada,

Scarlett Mac-Ginty, Jorge Gaete, & Daniel Núñez

**Supplemental Table 1.**

Frequency table of response categories by AAQ-II items.

| **AAQ-II Items** | **Response categories** | | | | | | |
| --- | --- | --- | --- | --- | --- | --- | --- |
|  | **Never** | **Very seldom** | **Seldom** | **Sometimes** | **Frequently** | **Almost always** | **Always** |
| AAQ1. My painful experiences and memories make it difficult for me to live a life that I would value. | 353 (23.5%) | 363 (24.2%) | 182 (12.1%) | 296 (19.7%) | 160 (10.6%) | 92 (6.1%) | 57 (3.8%) |
| AAQ2. I'm afraid of my feelings. | 297 (19.8%) | 275 (18.3%) | 195 (13.0%) | 332 (22.1%) | 207 (13.8%) | 134 (8.9%) | 63 (4.2%) |
| AAQ3. I worry about not being able to control my worries and feelings. | 214 (14.2%) | 237 (15.8%) | 185 (12.3%) | 307 (20.4%) | 248 (16.5%) | 183 (12.2%) | 129 (8.6%) |
| AAQ4. My painful memories prevent me from having a fulfilling life. | 525 (34.9%) | 326 (21.7%) | 177 (11.8%) | 207 (13.8%) | 116 (7.7%) | 77 (5.1%) | 75 (5.0%) |
| AAQ5. Emotions cause problems in my life. | 310 (20.6%) | 283 (18.8%) | 220 (14.6%) | 306 (20.4%) | 184 (12.2%) | 110 (7.3%) | 90 (6.0%) |
| AAQ6. It seems like most people are handling their lives better than I am. | 293 (19.5%) | 252 (16.8%) | 179 (11.9%) | 269 (17.9%) | 212 (14.1%) | 160 (10.6%) | 138 (9.2%) |
| AAQ7. Worries get in the way of my success. | 272 (18.1%) | 292 (19.4%) | 190 (12.6%) | 325 (21.6%) | 205 (13.6%) | 124 (8.3%) | 95 (6.3%) |

**Note.** AAQ-II: Acceptance and Action Questionnaire-II.

**Supplemental Table 2**

Discrimination (α_i_), and localization (β_ik_) parameters for AAQ-II items by gender groups

|  |  |  |  |  | **AAQ-II Items** |  |  |  |
| --- | --- | --- | --- | --- | --- | --- | --- | --- |
|  | **Parameters** | **AAQ1** | **AAQ2** | **AAQ3** | **AAQ4** | **AAQ5** | **AAQ6** | **AAQ7** |
| Group = Males | α_i_ | 2.657 (0.214) | 3.003 (0.231) | 3.121 (0.226) | 2.499 (0.214) | 3.558 (0.281) | 2.326 (0.173) | 2.898 (0.213) |
|  | β_i1_ | -0.727 (0.074) | -0.785 (0.073) | -0.901 (0.076) | -0.258 (0.067) | -0.718 (0.068) | -0.903 (0.083) | -0.844 (0.075) |
|  | β_i2_ | 0.118 (0.064) | -0.135 (0.062) | -0.296 (0.063) | 0.411 (0.069) | -0.104 (0.059) | -0.225 (0.067) | -0.176 (0.062) |
|  | β_i3_ | 0.539 (0.071) | 0.312 (0.064) | 0.121 (0.061) | 0.908 (0.085) | 0.353 (0.062) | 0.211 (0.068) | 0.270 (0.064) |
|  | β_i4_ | 1.308 (0.102) | 1.008 (0.082) | 0.763 (0.073) | 1.439 (0.115) | 1.036 (0.081) | 0.786 (0.079) | 0.888 (0.078) |
|  | β_i5_ | 2.097 (0.151) | 1.569 (0.112) | 1.342 (0.097) | 1.975 (0.153) | 1.624 (0.110) | 1.373 (0.105) | 1.564 (0.110) |
|  | β_i6_ | 2.692 (0.209) | 2.579 (0.190) | 1.977 (0.136) | 2.421 (0.189) | 2.133 (0.142) | 2.109 (0.157) | 2.165 (0.153) |
| Group = Females | α_i_ | 2.862 (0.158) | 2.886 (0.148) | 3.166 (0.158) | 2.974 (0.168) | 4.298 (0.224) | 2.543 (0.131) | 3.548 (0.179) |
|  | β_i1_ | -0.925 (0.055) | -1.094 (0.059) | -1.416 (0.066) | -0.573 (0.049) | -0.988 (0.051) | -1.141 (0.063) | -1.137 (0.056) |
|  | β_i2_ | 0.198 (0.064) | -0.467 (0.064) | -0.747 (0.049) | 0.071 (0.044) | -0.383 (0.042) | -0.529 (0.050) | -0.446 (0.045) |
|  | β_i3_ | 0.134 (0.071) | -0.119 (0.071) | -0.388 (0.045) | 0.384 (0.047) | -0.025 (0.041) | -0.182 (0.047) | -0.117 (0.043) |
|  | β_i4_ | 0.802 (0.102) | 0.575 (0.102) | 0.177 (0.044) | 0.913 (0.055) | 0.554 (0.045) | 0.354 (0.049) | 0.533 (0.046) |
|  | β_i5_ | 1.339 (0.151) | 1.173 (0.151) | 0.747 (0.051) | 1.322 (0.067) | 1.065 (0.053) | 0.847 (0.056) | 1.043 (0.055) |
|  | β_i6_ | 1.990 (0.209) | 1.864 (0.209) | 1.426 (0.068) | 1.806 (0.085) | 1.581 (0.068) | 1.435 (0.073) | 1.598 (0.072) |
| Uniform DIF | HQ | 15.331 | 18.347 | -0.937 | 16.654 | 20.020 | 21.563 | 14.006 |
|  | X^2^ | 8.548 | 5.532 | 24.817 | 7.226 | 3.859 | 2.316 | 9.873 |
|  | df | 6 | 6 | 6 | 6 | 6 | 6 | 6 |
|  | p-value | 0.201 | 0.478 | <0.001 | 0.301 | 0.696 | 0.888 | 0.130 |

**Note.** **Fit indices**: X^2^ =Chi-square statistic; df =degrees of freedom; DIF =Differential item functioning. Numbers in parentheses are standard error for estimates.
